# Promoting continence in nursing homes in four European countries: the use of PACES as a mechanism for improving the uptake of evidence-based recommendations

**DOI:** 10.1111/j.1744-1609.2012.00296.x

**Published:** 2012-11-23

**Authors:** Gill Harvey, Alison Kitson, Zachary Munn

**Affiliations:** 1 Health Management Group, Manchester Business SchoolManchester, UK; 2 School of Nursing, Royal Adelaide Hospital, University of AdelaideAdelaide, South Australia, Australia; 3 The Joanna Briggs Institute, Evidence Translation and Innovation, Royal Adelaide Hospital, University of AdelaideAdelaide, South Australia, Australia

**Keywords:** audit, continence care, evidence-based practice, facilitation, implementation, PACES

## Abstract

**Background:**

Multi-faceted approaches are generally recognised as the most effective way to support the implementation of evidence into practice. Audit and feedback often constitute one element of a multi-faceted implementation package, alongside other strategies, such as interactive education and facilitated support mechanisms. This paper describes a multi-faceted implementation strategy that used the Joanna Briggs Institute Practical Application of Clinical Evidence System (PACES) as an online audit tool to support facilitators working to introduce evidence-based continence recommendations in nursing homes in four different European countries.

**Aims/objectives:**

The paper describes the experience of using PACES with an international group of nursing home facilitators. In particular, the objectives of the paper are: to describe the process of introducing PACES to internal facilitators in eight nursing homes; to discuss the progress made during a 12-month period of collecting and analysing audit data using PACES; to summarise the collective experience of using PACES, including reflections on its strengths and limitations.

**Methods:**

Descriptive data were collected during the 12-month period of working with PACES in the eight nursing home sites. These data included digital and written notes taken at an initial 3-day introductory programme, at monthly teleconferences held between the external and internal facilitators and at a final 2-day meeting. Qualitative analysis of the data was undertaken on an ongoing basis throughout the implementation period, which enabled formative evaluation of PACES. A final summative evaluation of the experience of using PACES was undertaken as part of the closing project meeting in June 2011.

**Results:**

The nursing home facilitators took longer than anticipated to introduce PACES and it was only after 9–10 months that they became confident and comfortable using the system. This was due to a combination of factors, including a lack of audit knowledge and skills, limited IT access and skills, language difficulties and problems with the PACES system itself. The initial plan of undertaking a full baseline audit followed by focused action cycles had to be revised to allow a more staged, smaller-scale approach to implementation and audit. This involved simplifying the audit process and removing steps such as the calculation of population size estimates. As a result, an accurate baseline measure, prior to introducing changes to continence care, was not achieved. However, by the end of the 12 months, the majority of facilitators had undertaken a full audit and reported value in the process. In particular, they benefited from comparing audit data across sites to share learning and best practice.

**Discussion/conclusion:**

Working with PACES as part of a facilitated programme to support the implementation of evidence-based continence recommendations in nursing homes in four European countries has been a valuable learning experience, although not without its challenges. The findings highlight the importance of thorough training and support for first time users of PACES and the need to make the audit process as simple as possible in the initial stages.

## Background

The challenges of translating research evidence and clinical guidelines into practice are well recognised.^1^,^2^ From a starting point in the early days of evidence-based health care, where the implementation of evidence was largely conceptualised as a linear, rational process,^3^ it is now increasingly recognised that implementation is a complex, multi-faceted process, involving the interaction of numerous factors at the individual, team and wider organisational level.^4^ A number of models and frameworks have been developed that attempt to represent the complexity of the implementation process, for example: the Knowledge to Action cycle;^5^ the Joanna Briggs Institute (JBI) model of evidence based health care;^6^ and the Promoting Action on Research Implementation in Health Services (PARIHS) framework.^7^

The PARIHS framework is the focus of the study described in this paper. The framework proposes that the successful implementation of evidence into practice is dependent on the complex interplay of the evidence to be implemented (how robust it is and how it fits with clinical, patient and local experience),^8^ the local context in which implementation is to take place (the prevailing culture, leadership and commitment to evaluation and learning),^9^ and the way in which the process is facilitated (how and by whom).^10^ Since its initial publication, the PARIHS framework has been further refined^11^,^12^ and has been applied in practice, both as a heuristic to guide the application of research evidence into practice and as the conceptual underpinning of a variety of tools and measures.^13^ A recent critical synthesis of empirical studies in which the PARIHS framework had been used highlighted its strengths and weaknesses. In particular, the review concluded that there was a real need for the framework to be used prospectively in implementation studies.^13^

The Facilitating Implementation of Research Evidence (FIRE) study is one such example of a prospective study, with a particular focus on the facilitation dimension of the PARIHS framework. It is a four year, European Union funded study that aims to test two different models of facilitation against a standard method of disseminating evidence of best practice on continence promotion.^14^ The two different approaches to facilitation are labelled Type A and Type B and represent different points on the continuum of facilitation that was described in the PARIHS concept development papers.^10^ Type A facilitation is a pragmatic, quality improvement-based intervention, whilst Type B is an enabling, critical social science-based approach, with an emphasis on inquiry, reflection and emancipatory action. This paper focuses on the programme of work undertaken within the Type A facilitation intervention and, in particular, on the use of PACES as one of the elements within the facilitation component.

Before describing the use of PACES within the facilitation intervention, a brief overview of the FIRE study is provided, including the participating sites and countries and the evidence-based recommendations that are the focus of implementation efforts. The Type A facilitation intervention is then described in more detail, including the use of PACES as an audit and feedback mechanism within the overall facilitation package. The experiences of external and internal facilitators using PACES are presented and lessons learnt as a result of this experience are summarised.

### The FIRE study: an overview

The FIRE study commenced in January 2009 and involves 24 nursing homes from four European countries, namely, Sweden, England, Ireland and the Netherlands. Full details of the study protocol are published elsewhere.^14^ In summary, within each country, two nursing homes are randomly allocated to the control group (standard dissemination; evidence-based recommendations plus a PowerPoint presentation on implementation), two to Type A facilitation and two to Type B facilitation.^i^ In each group, the nursing homes have been asked to implement evidence-based recommendations on the management of incontinence in the frail elderly, developed by an international committee.^15^ In both the intervention groups (Type A and Type B facilitation), an internal facilitator was appointed from within the nursing home, using pre-determined selection criteria. All of the internal facilitators then attended a residential development programme in the Netherlands (one programme for Type A and one for Type B) with two external facilitators to prepare them for the role.

In the Type A facilitation group, two of the authors (GH and AK) acted as the external facilitators and designed the facilitation intervention. Following the three day residential programme, they then provided ongoing support to the internal facilitators via monthly teleconferences.

### Components of Type A facilitation

As noted earlier, Type A facilitation is described as a pragmatic approach to facilitating evidence into practice and focuses on addressing issues of implementation at the level of clinical teams, in terms of enabling them to design systems and processes of care that will enhance the transfer of evidence into their day to day practice. It draws on an eclectic range of theories, derived from management science, organisational learning, quality improvement and humanistic psychology. Individuals are prepared to take on the role of facilitator and are provided with a ‘toolkit’ of methods and techniques that they can use with health care teams to facilitate both the task and the process of implementing evidence.^16^ Thus, the initial development programme for internal facilitators from the eight nursing homes (two from each of the four countries) covered a range of topics, including understanding and interpreting evidence based recommendations in a local context, agreeing aims and planning for implementation, auditing and re-auditing practice, taking action to improve, the facilitator role and facilitation methods (see Box 1).

**Box 1 Key components of the Type A facilitator development programme**

**Table:** 

Topic area	Key areas covered
Interpreting the evidence	Introducing the evidence-based recommendations
	Thinking about the recommendations in the context of individual nursing homes
Planning for implementation	Agreeing aims and planning for local implementation
	Undertaking a baseline audit using PACES
	Taking action to improve using Plan-Do-Study-Act (PDSA) small tests of change
	Re-auditing and comparing audit data against baseline in PACES
Facilitation and the facilitator role	Purpose and attributes of the facilitator role
	Reflecting and building on own knowledge and skills
	Facilitation tools and methods
	Balancing task, team and individual needs

**Figure 1:**
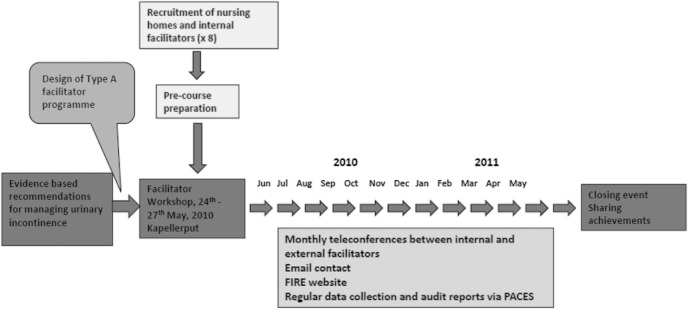
Summary of Type A facilitation.

To support the process of audit and feedback, the decision was made when planning the Type A intervention to use JBI PACES (the Practical Application of Clinical Evidence System), as this would provide an online tool that could be used to conduct audits within the individual nursing home settings. This was seen to fit well with the overall design of Type A facilitation, particularly as the ongoing support for internal facilitators would continue using virtual methods following the initial development programme (see Fig. 1). It is important to note that although PACES formed part of the Type A facilitator intervention, compliance to the evidence-based continence recommendations across all 24 sites in the FIRE study is assessed by independent researchers in each of the four countries, as part of the overall FIRE study protocol.^14^ The only data reported in this paper are the self-reported audit data collected by Type A facilitators using PACES.

## Aims and objectives

Having made the decision to adopt PACES as an audit and feedback system within the Type A facilitation component of the FIRE study, this paper aims to describe our experience of using PACES with an international group of nursing home facilitators. In particular, the objectives of the paper are as follows:

1. To describe the process of introducing PACES to internal facilitators in the eight nursing homes that were randomly allocated to Type A facilitation;
2. To present the progress made during the 12 months of the facilitation intervention in collecting and analysing audit data using PACES;
3. To summarise the internal and external facilitators' experiences of using PACES, including reflections on its strengths and limitations within the context of the Type A facilitation programme.

## Methods

Descriptive data were collected during the 12-month period of working with PACES in the eight nursing home sites. These data included digital and written notes taken at the initial 3-day development programme, at monthly teleconferences held between the external and internal facilitators and at a final 2-day meeting held in Stockholm at the end of the Type A facilitation intervention (June 2011). Qualitative analysis was undertaken by the two external facilitators (GH and AK) on an ongoing basis throughout the intervention period. For example, after each monthly teleconference, the notes from the meeting were transcribed and manually analysed by the two external facilitators. Important issues emerging from this analysis were discussed, reflected upon and noted and then fed into subsequent teleconference meetings. This enabled formative evaluation of PACES whilst the project was underway and any technical issues arising were discussed with the JBI contact for the project (ZM). A final summative evaluation of the experience of using PACES was undertaken as part of the closing project meeting in June 2011.

## Results

Presentation of the results is based around the three main objectives of the paper, namely: the process of introducing PACES within an international project to implement evidence into practice; progress made in applying PACES to audit evidence based recommendations on continence care; and reflections on the experience of using PACES in this way.

### The process of introducing PACES

**Figure 2:**
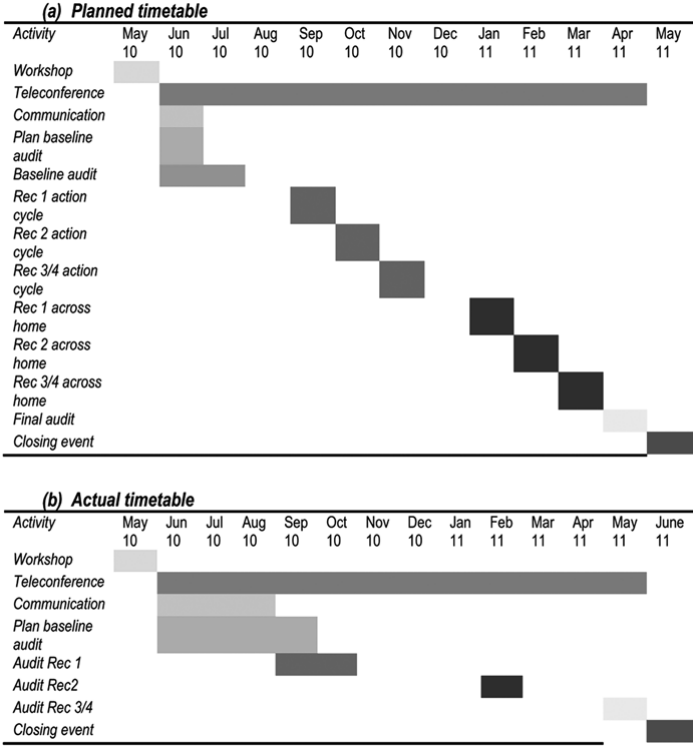
Audit and implementation timetable. (a) Planned timetable. (b) Actual timetable.

The starting point for developing the criteria to be included in the audit was an existing national audit tool for continence care,^17^ which corresponded well with the four recommendations. Audit criteria were drafted and agreed as part of one of the learning sessions at the residential programme, including agreeing on the level of compliance to be aimed for. In total, 16 audit criteria were developed as indicators of the four recommendations (see Box 2). At this same event, the PACES system was demonstrated to the internal facilitators and a discussion took place to plan a process and timetable for the audit exercise. As a result of this discussion, the decision was taken to commence with a full baseline audit of all 16 audit criteria, after which the facilitators would introduce action cycles for one recommendation at a time. It was felt that a staged process of implementing the evidence-based recommendations would make the process more manageable, particularly as for many of the internal facilitators, this would be their first experience of audit. A timetable was subsequently agreed that would enable each of the four recommendations to have a baseline audit, followed by an implementation/action plan, a period of spread to the wider nursing home, and re-audit, within the overall timescale of the study (see Fig. 2a).

**Box 2 PACES criteria for managing urinary incontinence in frail older peopleiii**

**Recommendation 1:** Each patient/resident has been actively screened for symptoms of urinary incontinence (UI)

Agreed Compliance: 100%

**Audit Criteria:**

1.1 Each resident has a documented record of their continence history
1.2 The continence history includes detailed information about the bladder habits of the resident
1.3 The continence history includes information about the resident's medical condition that may affect their ability to be continent

**Recommendation 2:** A detailed assessment is carried out which includes an assessment of co-morbid conditions, full urinalysis, wet checks to assess frequency and type of UI specified

Agreed Compliance: 100%

**Audit Criteria:**

2.1 Each resident who has been identified as having UI has a documented record of their continence assessment
2.2 The continence assessment includes a detailed consideration of relevant factors that might influence the resident's ability to be continent, both during the day and at night
2.3 The assessment includes a frequency volume chart
2.4 The assessment includes an indication of the type of UI

**Recommendation 3:** An individual treatment plan should be in place for setting individual goals for each resident with UI, working with residents' treatment preferences, explicit bladder retraining programs and linking the treatment plan to the resident's overall quality of life.

Agreed Compliance: 100%

**Audit Criteria:**

3.1 There is an individualised treatment plan for every resident with UI
3.2 The plan includes review dates
3.3 The plan is up to date at the time of audit
3.4 Relevant methods of management are documented as being used
3.5 There is documented evidence that the resident/representative has been involved in developing the plan of care for managing their urinary incontinence
3.6 There is documented evidence of active intervention for those residents where indicated in the assessment

**Recommendation 4**: Specialist referral should be made where required

Agreed Compliance: 100%

**Audit Criteria:**

4.1 Residents who require specialist referral get referred to the appropriate specialist
4.2 Residents' care plans document the reasons for the referral
4.3 Residents' care plans document the outcome of the referral

Following the residential programme, the two external facilitators worked with the JBI contact (ZM) to set up the continence audit within PACES, using the agreed criteria. A brief user guide for PACES was developed (ZM) and circulated to the internal facilitators, along with the offer of individual/technical support (from ZM and the two external facilitators) as and when needed. Each nursing home facilitator was provided with login details to access PACES for the purposes of inputting audit data and viewing results.

All sites were to start with a baseline audit of all the recommendations and were initially asked to make an estimate of compliance to the audit criteria, so that the JBI administrator (ZM) could calculate the sample size each home needed to work with. For many of the internal facilitators, this step in the process caused some confusion. Some were unable to distinguish the process of estimating compliance from the actual collection of audit data, leading to misunderstanding and different interpretations of what they were being asked to do. This was further compounded by a number of practical and logistical difficulties experienced by some of the internal facilitators, including language barriers, limited IT access and skills and unfamiliarity with audit. This delayed the process of starting the baseline audit proper, as additional time had to be spent discussing what estimating the compliance entailed and, in some cases, providing additional practical support to calculate the estimate.

### Progress in applying PACES to audit evidence-based continence recommendations on continence care

Progress with audit was a regular agenda item at the monthly teleconferences and for most of these, the JBI contact (ZM) joined for part of the call to answer any audit queries that had arisen and talk through any difficulties the internal facilitators were experiencing. Box 3 provides a narrative summary of the progress made in using PACES over the 12 months of the project and issues that arose during this time.

**Box 3 Narrative summary of progress in introducing PACES over a 12-month period**

**Table:** 

Teleconference	Discussion on using PACES
1	Criteria translated and uploaded into PACES; homes asked to calculate population size compliance estimate
June 2010	Some facilitators not sure how to collect audit data
	Two facilitators said they would prefer to audit recommendation 1 only to make it more manageable
	For consistency, agreed that everyone should start with recommendation 1: a baseline audit, followed by an action cycle to increase compliance; aim to complete by September 2010
2	Some facilitators had calculated compliance rates, in preparation for baseline audit; others not yet at this stage
July 2010	Several already starting to think about changes that they wanted to introduce
3	Baseline audit ready to commence in PACES; facilitators reported problems in trying to access PACES
August 2010	JBI contact offered additional help to guide facilitators through the process of logging on to PACES
	One facilitator asked to do a full baseline audit of all four recommendations; agreed this was possible, but would have to be set up separately in PACES
	Agreed a new deadline of the end of September to enter audit data for recommendation 1 audit criteria
4	Plan had been to have completed baseline audit for recommendation 1, but not achieved; facilitators still experiencing problems
September 2010	Some confused by the step of estimating compliance and had actually collected audit data to estimate compliance; others had used a different sample size than their original compliance estimate
	JBI contact offered to enter data into PACES if facilitators collected it using a paper-based system
5	4 sets of audit data for recommendation 1 received
October 2010	Some facilitators had fed back data to colleagues and managers; some starting to introduce changes using PDSA cycles
	Reflective discussion on learning from audit of recommendation 1: initial calculation of compliance estimates, as the basis for setting sample sizes, was difficult and some homes interpreted this as the audit proper; problems with some nursing homes not having computer access
	Agreed that external facilitators and JBI contact would specify sample sizes for audit of recommendation 2
6	Sample sizes for audit of recommendation 2 confirmed, ready to be uploaded into PACES
	November 2010
7	Still waiting to get underway with the audit of recommendation 2
	December 2010
8	General review of progress made to date; sense that project is challenging, but a lot of work underway to communicate with colleagues and introduce changes to improve the management of continence
	January 2011
9	Audit data submitted for recommendation 2 for 7 of the 8 nursing homes; agreed to share data across homes
February 2011	Facilitators report feeling more comfortable using the PACES programme
	Planning actions relating to the assessment of continence
10	Helpful to look at own results in relation to other sites; can recognise where more work needs to be done
March 2011	Recognising benefits of having audit data
	PACES proving helpful; know exactly what data have to be collected; a very organised way of working
	All facilitators moving on to think about the audit of recommendation 3
11	Agreed to use the same sample size as for audit of recommendation 2 to simplify the process
April 2011	Different homes at different stages of readiness for audit of recommendation 3, although most already involved in action e.g. introducing new documentation for care plans
12	Most facilitators in process of auditing recommendation 3; some also planning for recommendation 4
May 2011	Discussion about what audit is actually measuring at this point in time; not a true measure of baseline as most homes have already started to introduce changes since initial audits of recommendation 1 and 2 were undertaken

Within the first few months, a number of specific difficulties and challenges became apparent. Aside from the initial problems in calculating compliance estimates, a lack of confidence and skills in using computers (and in one case, a lack of access to a computer within the nursing home) posed a significant challenge to some of the internal facilitators. For this reason, it was agreed that facilitators could collect audit data using a paper-based system and forward the results for entry into PACES. This was an offer that the majority of the internal facilitators took up.

Despite the plans made at the residential programme, it also became clear that for many homes, the work needed in the initial stages of implementation to inform colleagues, managers, residents and relatives about the project and what it would involve took a lot longer than anticipated. This was particularly the case where facilitators had to do a lot of work to get support for the project and convince people to get involved. All of the facilitators had been asked to identify a buddy to work alongside and support them, and it was suggested that they set up a project team to lead the work in implementing and auditing the recommendations. In practice, some facilitators did set up this collaborative approach to the project; others tended to do most of the work on their own. However, the net result of the effort required to get started and elicit interest and support for the project was to delay the start of the internal facilitators' baseline audit.

This dual challenge of limited knowledge and skills in audit and the additional time needed to get things started left some facilitators feeling overwhelmed by the prospect of undertaking a full baseline audit of all 16 criteria. As early as the first teleconference, two of the facilitators expressed concerns and said they would prefer to start with an audit of recommendation 1 only. In order to maintain a uniform approach across all of the nursing homes, it was then agreed that all the facilitators should proceed in this way, thus applying a staged approach to both audit and implementation (see Fig. 2b). However, an unintended consequence of this decision was that once homes starting looking at the audit of the first recommendation (screening for problems with continence), they immediately started to act on their audit findings and introduce more comprehensive screening and assessment methods. The impact of this was that it was difficult to get a true baseline measure of continence care, according to the four evidence-based recommendations. This was particularly so for recommendations 2–4, which were meant to be addressed in a sequential way, according to the revised audit timetable. However, in practice, this staged approach to audit and re-audit did not work as planned. Once the nursing homes started looking at the recommendations and how they performed against the first one, most of them began to introduce changes (for example, in the form of new documentation and educational sessions for staff), which encompassed elements of the audit criteria for recommendations 2 to 4.

The combination of these various issues that arose during the process of audit and implementation was that the planned approach working from recommendation 1 to recommendation 4 over a 12-month period did not work in practice. By the end of month 5, the majority of homes^ii^had collected audit data for the first recommendation and were ready to move onto auditing the subsequent recommendations. From this point, individual homes tended to work at their own pace and by the end of the 12-month intervention period, seven of the eight homes had successfully used PACES to collect audit data on continence care. All of these seven homes had collected data on recommendations 1 and 2; five homes had audited recommendation 3; four homes had also collected audit data on recommendation 4 and had, therefore, successfully audited all four of the evidence-based recommendations (see Table 1). None of the homes had got to the point of completing a re-audit.

**Table 1:** Summary of progress in applying PACES

Evidence-based recommendation	Number of homes who completed audit	Average self-reported range of compliance (%)
1	7	10–100
2	7	15–97
3	5	12–100
4	4	9–100

As previously noted, the audit data reflect a point in the process of implementation, rather than a true baseline measure. In terms of the overall project, however, the audit data, although imperfect from a measurement sense, became an extremely valuable tool within the teleconference discussions, as the narrative in Box 3 illustrates. There was considerable variation in the self-reported rates of achieving the different audit criteria, ranging from 0 to 100% (Table 1). Within the teleconferences, the external facilitators were able to use the comparative data to facilitate information sharing amongst internal facilitators, particularly in terms of encouraging homes with high levels of compliance to share what they had done to achieve these results. On a number of different occasions, this acted as a catalyst for further information exchange amongst the group, for example, in terms of sharing written documentation or educational resources that they had developed to support implementation.

## Reflections on the experience of using PACES

At the close of project meeting, the external facilitators led a session in which internal facilitators were asked to identify what worked well during the project, areas of challenge, areas of learning and what they would do differently if starting again. The two external facilitators also completed a similar process of reflection. In relation to the introduction and use of PACES, a number of issues emerged. Firstly, the ‘newness’ of many of the internal facilitators to the role meant that they were trying to learn a lot of new skills all at the same time. For those who had no or little previous experience of audit, the prospect of using an online audit tool such as PACES was quite daunting. This was exacerbated where internal facilitators had limited IT access or skills and where language barriers proved problematic, for example, in terms of written documentation in a second language or following all of the discussion at the monthly teleconferences. Despite these difficulties, the availability of audit data that they could use to demonstrate progress with implementation was valued by the internal facilitators.

Interestingly, the internal facilitators did not share the external facilitators' concerns about the lack of a true baseline measure within the audit process. From the external facilitators' perspective, this meant that it was difficult to judge more objectively what progress was made within individual homes over time, particularly as the only contact with the homes was via virtual media. In reflecting on how things might have been done differently, both of the external facilitators felt that it would have been better to start with a full baseline audit of all the recommendations as originally planned, rather than the staged approach that was adopted.

More specifically in terms of using PACES, two points that arose from the external facilitators' reflections in their role as coordinators of the audit process related to the confusion caused by estimating compliance and the inability to produce comparative data across all the sites within PACES. On the first point of calculating compliance estimates, this appeared to be too complicated a step in the initial process of introducing PACES for the novice facilitators within this study and, in hindsight, it may have been better to omit this step (e.g. by simply giving each home a sample size to work with from the outset). At this initial stage, it was important to make the internal facilitators feel confident and comfortable with using PACES, rather than getting the best possible audit data. Linked to this point, one of the most valuable uses of the audit data was comparing and discussing achievement rates across the different nursing homes. However, comparison of the data in this way could not be done within PACES. Whilst PACES allows comparison of results within an individual site, comparison across multiple sites (such as in the FIRE study) had to be undertaken using an alternative data package (in this case, Excel).

## Discussion

Working with PACES within the context of an international study has been a valuable learning experience. Both the international nature of the project and the setting in which the evidence-based recommendations were implemented presented challenges that had to be overcome. For example, half of the facilitators were working with English as their second language, which meant that written documentation relating to the audit had to be translated where possible. The nursing home setting posed particular challenges in that some facilitators had limited computer access within the nursing home, which made the use of a Web-based audit system such as PACES very difficult. Even where IT access was not a problem, a number of the facilitators in the project had limited IT skills, which caused difficulty in accessing and using PACES in the early stages of the project. This was compounded by the fact that the facilitators had only a short introduction to PACES in the initial residential programme, after which all contact was by virtual methods. As the data illustrate, it was not until months 9 and 10 that the facilitators started to feel comfortable using PACES and could see the benefits of the system. This has implications for introducing PACES to a similar group of users in the future, in terms of anticipating the level of IT skills and basic audit knowledge and skills required to use the system to full effect. Without ongoing support from the external facilitators and the JBI contact, the internal facilitators in the nursing homes would have found it extremely difficult to understand and use PACES effectively.

What became increasingly clear as the project progressed was the need to simplify the audit process as much as possible. This included removing the step where the nursing homes were asked to calculate population size compliance estimates, as this caused confusion at a stage in the process where many facilitators were still coming to terms with the basic principles of audit. Another measure taken to simplify the audit process was to stage the data collection, rather than starting with a full baseline audit as originally planned. In hindsight, this may not have been the best decision as it meant that there was a not a true baseline measure of where the homes started from. However, this proved to be more of a concern to the external facilitators than the internal facilitators, the latter group being more focused on using data to guide and focus improvements, rather than getting an ‘absolute’ measure of where they started from. This raises an interesting question about how much pressure to put on clinical teams to get the audit process right from the beginning or whether to let them have a go and learn through experience, as was the case in this project. The answer to the question probably depends on the primary purpose of audit; in this case, the emphasis was on developing audit skills as a foundation for quality improvement, something which required a considerable amount of time and support. Although an accurate baseline measure of practice before any changes were introduced would have been ideal, in practice this was seen to be too demanding a requirement for novice facilitators with little prior experience of audit and no previous experience of using an electronic data capture system. Consequently, the decision was taken to scale back the initial introduction of PACES to match the stage of learning and development of the facilitators.

The experiences of the facilitators in this study reflect the complexities of the audit process and the need to develop the technical skills in audit at the same time as creating the right environment for audit.^18^ One of the most powerful ways that audit data were used to guide improvement was through comparing data across sites, in order to share learning and pass on best practice. Presenting the data in a way that allowed each nursing home to view their own results alongside those of the other homes proved to be a powerful learning tool, as other studies have also demonstrated.^19^ This could be a useful point for consideration in any future development of the PACES system.

## Conclusions

This paper has discussed the use of PACES as part of a facilitated programme to support the implementation of evidence-based continence recommendations in nursing homes in four European countries. The findings highlight that audit skills are an important foundation for quality improvement, but do not necessarily come easily or automatically to clinical staff. The facilitators in this study required a considerable amount of time and support to become comfortable in using PACES and would have benefited from a ‘hands-on’ opportunity to use the system during the initial facilitator development programme. The need for accurate baseline data had to be balanced against developing skills and confidence in collecting audit data and using PACES. However, by the end of a 12-month period, the majority of the facilitators had successfully audited all of the evidence-based recommendations and introduced changes to improve patient care.

The study also highlights some potentially useful learning points in relation to the future development and application of PACES (see Box 4). These include consideration of the training and support needs of novice facilitators and audit teams when working with PACES, clearer guidance on when and how to use population size compliance estimates and providing a facility within the data analysis component of PACES to compare data across multiple sites.

**Box 4 Key learning points in relation to using PACES**

- First time users of PACES need training in the use of the system
- This training needs to be tailored to the individuals/teams that will be using the system in terms of their level of audit knowledge, skills and experience and any specific language requirements
- First time users should be advised to conduct smaller scale audits without sample size analysis so that they can acquaint themselves with the system
- PACES users should have access to a JBI contact for assistance where needed, particularly if their main contact with the system (or wider project, as in the case of the FIRE study) is virtual
- A facility for comparing data across sites would be a useful feature within the PACES system

## Footnotes

i Ethical Committee approval for the study was obtained in England, Sweden, and Republic of Ireland. In the Netherlands, the researchers were advised to get permission from either an ethical committee at site level, or where this did not exist, from a scientific or residents committee at the site. Research Governance approval was also obtained in England, and permission to collect data at the sites obtained in Sweden and Republic of Ireland.

ii One home did not submit any audit data during the project period; a second home experienced some delay as the original facilitator had to be replaced due to illness.

iii Developed and agreed by the internal and external facilitators from the original evidence-based continence recommendations.
